# *Bolanthus turcicus* (Caryophyllaceae), a new species from Turkey

**DOI:** 10.3897/phytokeys.52.4479

**Published:** 2015-07-08

**Authors:** Murat Koç, Ergin Hamzaoğlu

**Affiliations:** 1 Department of Biology, Faculty of Art & Sciences, Bozok University, 66100, Yozgat – Turkey; 2 Animal Production High School, Bozok University, 66100, Yozgat – Turkey; 3 Department of Elementary Education, Gazi Faculty of Education, Gazi University, 06500, Ankara - Turkey

**Keywords:** Anatolia, new species, *Bolanthus*, taxonomy

## Abstract

A new species *Bolanthus turcicus* Koç & Hamzaoğlu, **sp. nov.** was discovered on Hasan Mountain (Turkey, Aksaray province) where it grows on volcanic stony slopes and alpine steppe. its description, images, chorology and ecology, and threat category are provided in this article. It was compared with a closely related species, *Bolanthus minuartioides* (Jaub. & Spach) Hub.-Mor., *Bolanthus huber-morathii* C.Simon, *Bolanthus spergulifolius* (Jaub. & Spach) Hub.-Mor., *Bolanthus frankenioides* (Boiss) Bark., *Bolanthus mevlanae* Aytaç based on its general morphology and seed micromorphology.

## Introduction

*Bolanthus* (Ser.) Reichb., in the family Caryophyllaceae, is one of the smallest genera of the family. This genus resembles especially the species of *Gypsophila* L. and *Acanthophyllum* C.A.Mey by its physical appearance. Nevertheless, it differs from *Gypsophila* in that *Bolanthus* are calyx tubular and do not include druses. Besides, it is different from the species of *Acanthophyllum* as its fruit is 8–28-seeded, dehiscing by valves or teeth, leaves, bracts, and calyx never spiny, stamens included calyx. In addition, *Bolanthus* basically spreads across the Meditarranean Region, while *Acanthophyllum* is an Irano-Turanian genus (Bittrich 1993, Huber-Morath 1967, Huber-Morath et al. 1968, Davis et al. 1988).

*Bolanthus* consists of approximately 15 species and is mainly distributed in Greece, Palestine and Turkey (Bittrich 1993, Huber-Morath 1967, Davis et al. 1988). *Bolanthus* includes six species one of which is represented by 2 varieties in Flora of Turkey (Huber-Morath 1967, Huber-Morath et al. 1968). *Bolanthus* is represented by 8 species in Flora Europaea (Barkoudah and Akeroyd 1993), 1 species in Flora Palaestina (Zohary 1966). As a result of recent studies, 2 species (*Bolanthus huber-morathii* C.Simon, *Bolanthus mevlanae* Aytaç) and 1 subspecies (Bolanthus creutzburgii Greuter subsp. zaffranii Phitos, Turland & Bergmeier) have been added to this genus (Aytaç and Duman 2004, Özhatay et al. 2009, Phitos et al. 2011). As a result; the total taxa number of this genus has been increased to 20. Anatolia is a prominent centre for *Bolanthus* and 8 species grow in Turkey.

## Materials and methods

We came across some interesting *Bolanthus* specimens while conducting field work on the Hasan Mountain above Karkın town (Turkey, Aksaray province), as two authors actually having the goal of finding the *Minuartia* L. and *Dianthus* L.. These specimens were compared with related species in the herbarium of Biology Department of Bozok University, GAZI, K and with records in the literature (Barkoudah and Akeroyd 1993, Zohary 1966, Huber-Morath 1967, Huber-Morath et al. 1968, Davis et al. 1988, Bojňanský and Fargašová 2007). The images were taken using the Canon EOS 60D digital camera, and the seed surface micromorphology was visualized using the LEO 440 scanning electron microscope. Normal visualization of the specimens was carried out using the Olympus SZ61 microscope. The vegetative characters were measured using a ruler with 0.5-mm accuracy and the floral characters were determined using an ocular micrometer.

## Taxonomic treatment

### Bolanthus turcicus

Taxon classificationPlantaeCaryophyllalesCaryophyllaceae

Koç & Hamzaoğlu sp. nov.

urn:lsid:ipni.org:names:77148111-1

Figs 1 , 2

#### Diagnosis.

*Bolanthus turcicus* is related to *Bolanthus spergulifolius* (Jaub. & Spach) Hub.-Mor. It differs from the related taxa mainly by having it has leaves 3-veined (not 1-vained), linear (not subulate); calyx 3.5–4.5 mm long (not 4.5–5.5 mm long); petals 3.3–4.5 mm long and as long as calyx (not 5.5–6.5 mm long and 1.5 times longer than calyx).

#### Type.

TURKEY, Aksaray province, Hasan Mountain above Karkın town, Hamzaoğlu 7110 and Koç (holo GAZI, iso GAZI, ANK, Dept of Bozok Univ., Herbarium of Biology), 1950 m, volcanic stony slopes and alpine steppe, 18 June 2014.

#### Description.

Perennial, completely glandular and eglandular hairs. Stems prostrate, 8–14 cm tall, 0.5–1 mm diameter. Leaves linear, margins ciliate and subscarious near base, apex acute; sterile shoot leaves similar but shorter than or equal to cauline leaves; cauline leaves linear, 5-8 × 0.5-0.7 mm, 3-veined, sheaths equal or slightly longer than wide; upper similar but smaller. Inflorescence cymose, subcapitate, 5–10-flowered; bracts similar cauline leaves, 3–5 × 0.4–0.6 mm; ± equalling calyx; pedicels 1–2 mm or absent. Calyx tubular, 3.5–4.5 × 1–1.4 mm; tube 5-ribbed, herbaceous, commissures whitish, scarious; teeth narrowly triangular, 1–1.3 mm, apex acuminate. Petals linear-spathulate, 3.3–4.5 × 0.8–1.2 mm, emarginated, as long as calyx; limb whitish with transverse deep purple stripe near middle or base. Stamens 10. Styles 2. Capsule sessile, oblong-ovoid, including calyx, dehiscing by 4 teeth. Seeds black, comma-shaped, 0.8–1.1 × 0.5–0.7 mm, tuberculate. flowering in June and July, volcanic stony slopes and alpine steppe.

#### Seed micro-morphology.

Seeds of *Bolanthus turcicus* are comma-shaped, 0.8–1.1 × 0.5–0.7 mm, granular; ventral surface with regular elongated rectangular cells, tuberculate, obscure teeth on each margin, teeth S-undulate; dorsal surface with regular rectangular cells, tuberculate, obscure teeth on each margin, teeth S-undulate. The seeds of *Bolanthus turcicus* aren’t different than the seeds of *Bolanthus minuartioides* (Jaub. & Spach) Hub.-Mor., *Bolanthus huber-morathii*, *Bolanthus spergulifolius*, *Bolanthus frankenioides* (Boiss) Bark., *Bolanthus mevlanae* in terms of dorsal and ventral surfaces (Figure 2).

#### Chorology and ecology.

*Bolanthus turcicus* grows on volcanic stony slopes and alpine steppe and Irano-Turanian phytogeographic regions (Davis 1965). Many plants in the alpine steppe have distinctive adaptations to the harsh environment. On mountain slopes in the alpine steppe, grasses decrease and forbs increase (Huang 1987). The species grows on volcanic stony slopes and alpine steppe together with Scleranthus annus L. subsp. polycarpos (L.) Thell., *Acantholimon acerosum* (Willd.) Boiss., *Bromus tomentellus* Boiss., *Festuca valesiaca* Schleich., Alyssum pateri Nyár. subsp. pateri, *Astragalus angustifolius* Lam., *Astragalus lineatus* Lam., *Phlomis armeniaca* Willd., *Phleum alpinum* L., *Stipa pulcherrima* K.Koch, *Minuartia juniperina* (L.) Maire & Petitm., *Sedum pallidum* M.Bieb.

#### Conservation status.

*Bolanthus turcicus* is an endemic species known only from the type gathered in the Hasan Mountain (Aksaray province). Informal grazing and land-use changes could have a detrimental impact in the future. For this reason this species should be classified as “Critically Endangered” (CR-B1a) according to the World Conservation Union categories (IUCN 2014).

## Results

The specimens introduced as the new species in this study were collected from Aksaray province, Hasan Mountain above Karkın town. All the 20 taxa, which spreads across the world as well, exist in the Meditarranean Region; however, this taxon exists in the Irano-Turanian phytogeographic region. Firstly collected specimens resemble *Bolanthus spergulifolius* and *Bolanthus huber-morathii* at first glance. Yet, comprehensive studies that were subsequently carried out revealed that they belonged to a new species.

## Distinction from other taxa

**Figure 1.**
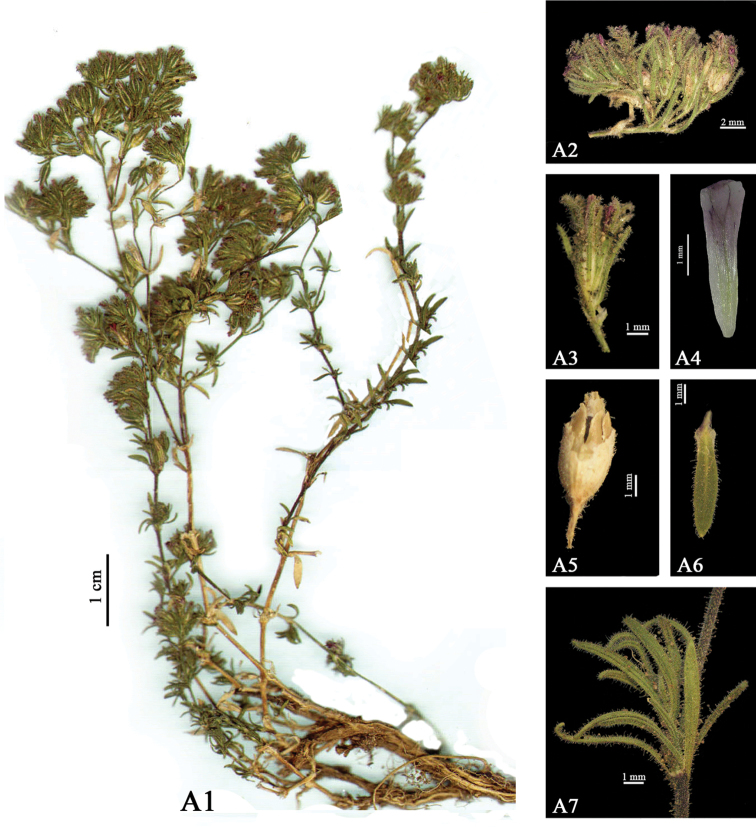
A: *Bolanthus turcicus* (EH 7110). **1** Habit **2** Inflorescence **3** Flower **4** Petal **5** Capsule **6** Leaf **7** Leaf axillary fascicles.

*Bolanthus* is represented by 8 species in the flora in Turkey. Three species (*Bolanthus cherlerioides* (Bornm.) Bark., *Bolanthus thymoides* Hub.-Mor., *Bolanthus stenopetalus* Hartving & Strid) among them differs from the others as these three have tight cushion-shaped, leaves that have intensive imbricates and their internodes are scarcely visible. Therefore, *Bolanthus turcicus* is similar to the other 5 species (*Bolanthus minuartioides*, *Bolanthus huber-morathii*, *Bolanthus spergulifolius*, *Bolanthus frankenioides*, *Bolanthus mevlanae*) as it branches loosely, its leaves do not imbricate and the gaps between the internodes are wide. Each of these species has its own character by its pedal color, shape of leaves and pubescence. According to the examined specimens and the Flora of Turkey and the East Aegean Islands, *Bolanthus turcicus* differs from the species of *Bolanthus frankenioides* and *Bolanthus mevlanae* with its loose branching, wide distance internodes, shapes and vein number of leaves, length of petal and ratio of petal-calyx. The increases in differences suspend it from these taxa while approximating to *Bolanthus minuartioides*, *Bolanthus spergulifolius*, *Bolanthus huber-morathii*. However, *Bolanthus huber-morathii* is a distinctive species, whose its glabrous stems, shape of leaves, rate of bracts to calyx and loose inflorescence,. On the other hand, *Bolanthus minuartioides* is a distinctive one as well with its petal colour, length of calyx-teeth and inflorescence shape. Thus *Bolanthus turcicus* resembles *Bolanthus spergulifolius* with its stems hairy, length of leaves, rate of bracts-calyx, type of inflorescence and color of petal. Despite these similarities, there are several distinctive differences between *Bolanthus turcicus* and *Bolanthus spergulifolius* in terms of leaves vein number, shape of leaves, rate of bracts-calyx, length of calyx, length of petal, rate of petal-calyx. A key-hart showing the discrimination between the related sprecies and tale showing the characteristics of the species are provided below. (Table 1, Figure 1).

**Figure 2.**
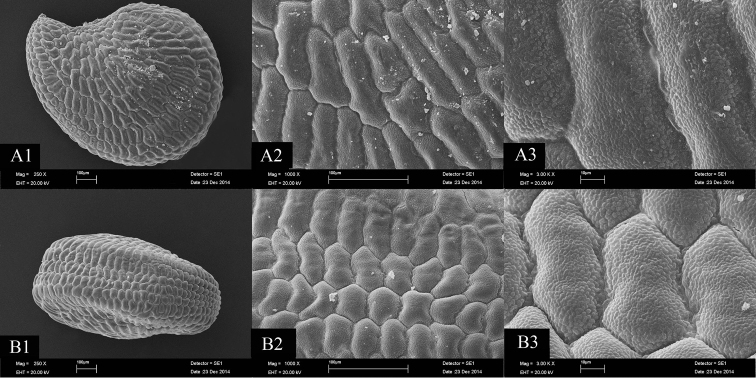
SEM photographs of the seed coat. *Bolanthus turcicus* – **A1–3** ventral surface **B1–3** dorsal surface (Scale bars 100 μm).

**Table 1.** Diagnostic characters *Bolanthus frankenioides*, *Bolanthus mevlanae*, *Bolanthus huber-morathii*, *Bolanthus minuartioides*, *Bolanthus spergulifolius* and *Bolanthus turcicus*.

**Characters**	***Bolanthus frankenioides***	***Bolanthus mevlanae***	***Bolanthus huber-morathii***	***Bolanthus minuartioides***	***Bolanthus spergulifolius***	***Bolanthus turcicus***
**Plants**	loosely tufted	loosely tufted	many branched	many branched	many branched	many branched
**Stems**	2–15 cm	3–5 cm	8–15 cm	3–15 cm	5–15 cm	8–14 cm
	densely covered with glandular and eglandular hairs	puberulent	glabrous	long eglandular hairs	densely glandular and eglandular hairs	densely glandular and eglandular hairs
**Leaves**	2–7 mm	2–5 mm	5–8 mm	3–7 mm	5–10 mm	6–10 mm
	linear-setaceous	setaceous	subulate-setaceous	subulate	subulate	linear
	1-veined	1-veined	1-veined	1-veined	1-veined	3-veined
**Bracts**	2/3 or as long as calyx	2/3 or as long as calyx	up to 1/2 as long as calyx	2/3 or as long as calyx	2/3 or as long as calyx	2/3 or as long as calyx
**Flowers**	1–3-flowered	5–10-flowered	5–15-flowered	(5-)10–25-flowered	5–15-flowered	5–10-flowered
**Inflorescence**	Solitary in the axils or loose few flowered dichasial	dense dichasial subcapitate subsessil clusters	lax dichasial clusters	dense dichasial subcapitate subsessil clusters	dense dichasial subcapitate subsessil clusters	dense dichasial subcapitate subsessil clusters
**Calyx**	3–3.5 mm	4–5 mm	4.5–6 mm	4–5 mm	4.5–5.5 mm	3.5–4.5 mm
**Calyx teeth**	0.5–0.7 mm	1.5–2 mm	0.7–1 mm	0.7–1 mm	1–1.5 mm	1–1.5 mm
**Petals**	linear-cuneate	linear-oblong	linear-spatulate	linear-lanceolate	linear-oblong	linear-spatulate
	5–7 mm	5.5–6 mm	7–8 mm	5–7 mm	6.5 mm	3.3–4.5 mm
	white with purple veins	white with purple veins	white with purple veins	white without purple veins	white with purple veins	white with purple veins
	1.5 times longer than calyx	1.5 times longer than calyx	1.5 times longer than calyx	1.5 times longer than calyx	1.5 times longer than calyx	as long as calyx
**Habitat**	alpine meadows	steppe	serpantine	dry hills, limestone, mountain steppe	steppe, stony places fields	volcanic stony slopes and alpine steppe

### Key to closely related *Bolanthus* species

**Table:** 

1	Plants loosely tufted; leaves setaceous or linear-setaceous, internodes 1–5 mm	
2	Stems densely covered with glandular and eglandular hairs, leaves linear- setaceous; calyx 3–3.5 mm, teeth 0.5–0.7 mm; inflorescence Solitary in the axils or loose few flowered dichasial.	***Bolanthus frankenioides***
2'	Stems puberulent, leaves setaceous; calyx 4–5 mm, teeth 1–1.5 mm; inflorescence dense dichasial subcapitate subsessil clusters	***Bolanthus mevlanae***
1'	Plants many branched, not tufted; leaves subulate, subulat-setaceous or linear, internodes 5–20 mm	
3	Stems glabrous; bracts up to 1/2 as long as calyx; inflorescence lax dichasial clusters	***Bolanthus huber-morathii***
3'	Stems glandular and eglandular hairy; bracts 2/3 or as long as calyx; inflorescence dense dichasial subcapitate subsessil clusters	
4	Stems long eglandular hairs; calyx teeth 0.7–1 mm; petals white without purple veins.	***Bolanthus minuartioides***
4'	Stems densely glandular and eglandular hairs; calyx teeth 1–1.5 mm; petals white with purple veins	
5	Leaves linear, 3-vained; calyx 3.5–4.5 mm long; petals 3.3–4.5 mm long, as long as calyx	***Bolanthus turcicus***
5'	Leaves subulate, 1-vained; calyx 4.5–5.5 mm long; petals 5.5–6.5 mm long, 1.5 times longer than calyx	***Bolanthus spergulifolius***

### Specimens examined

TURKEY - ***Bolanthus mevlanae***, C3 Antalya: Between Akseki and Bozkır, 45 km, Gölcük, 1780 m, 16.07.1997, Aytaç 7733 (GAZI!); C3 Antalya: Between Bozkır and Akören, 1100 m, 18.06.2013, Hamzaoğlu 6765 and Koç (Bozok Univ. Herb.) - ***Bolanthus huber-morathii***, A2 Bursa: Soğukpınar-Keles 1 km nach soğukpınar, 900 m, 08.07.1979 (GAZI!); B2 Kütahya: Tavşanlı district, around Arifler town, Koç 1757 and Kocakaya (Bozok Univ. Herb.) - ***Bolanthus spergulifolius***, B2 Uşak: Alma Dagh, N of Uşak, 1300 m, 1857, Balansa 1294 (K-000725786 photo!) - ***Bolanthus minuartioides***, C3 Konya: Derebucak, Çamlık town, 1300 m, 30.05.2005, Hamzaoğlu 3640 (Bozok Univ. Herb.); C3 Isparta: Keçiborlu, NW of Keçiborlu, 1250 m, 16.06.2013 Hamzaoğlu 6748 and Koç (Bozok Univ. Herb.); B3 Afyon: between İscehisar and Seydiler, 1150 m, 30.06.2012, Hamzaoğlu 6394 and Koç (Bozok Univ. Herb.); B3 Afyon: between İscehisar and Seydiler, 1150 m, 30.06.2012, Hamzaoğlu 6394 and Koç (Bozok Univ. Herb.) - ***Bolanthus frankenioides***, B2 Kütahya: İscehisar, 1150 m, 05.08.2012, Hamzaoğlu 6585 and Koç (Bozok Univ. Herb.!); B3 Afyonkarahisar: between Bayat and Iscehisar, 1500 m, 01.07.2010, Koç 1209 and Hamzaoğlu (Bozok Univ. Herb.!).

## Supplementary Material

XML Treatment for Bolanthus turcicus
